# A Diagnosis Method for Noise and Intermittent Faults in Analog Circuits Based on the Fusion of Multiscale Fuzzy Entropy Features and Amplitude Features

**DOI:** 10.3390/s25041090

**Published:** 2025-02-12

**Authors:** Junyou Shi, Yilei Hou, Zili Wang, Zhilin Yang, Zhenyang Lv

**Affiliations:** School of Reliability and Systems Engineering, Beihang University, Beijing 100191, China; shijy@buaa.edu.cn (J.S.); wzl@buaa.edu.cn (Z.W.); yangzl@buaa.edu.cn (Z.Y.); lzy@buaa.edu.cn (Z.L.)

**Keywords:** intermittent fault diagnosis, multiscale fuzzy entropy, amplitude features, analog circuits, noise

## Abstract

Intermittent faults occur randomly, last for short durations, and ultimately lead to permanent failures, threatening the safety and stability of analog circuits. Additionally, these faults are often hard to differentiate from noise-induced anomalies, resulting in incorrect disassembly and complicating circuit maintenance. To address these challenges, we propose a novel fault diagnosis method. The method uses an adjustable sliding window to extract multiscale fuzzy entropy features, mitigating the impact of normal data on entropy calculations for intermittent faults. The coarse granulation strategy of sliding point by point is applied to avoid information loss in short time series. The raw signal is then segmented and transformed into four statistical features, which are fused into comprehensive amplitude features via a self-attention mechanism. This comprehensive feature better captures amplitude variations than individual statistical features. Finally, the two features are fed into a convolutional neural network for diagnosis. The method is applied to two typical analog circuits. Ablation studies confirmed its effectiveness. Although the proposed method does not have the lowest diagnostic cost and the fastest detection time, the differences with state-of-the-art methods are minimal, and the proposed method achieves higher classification accuracy. Taken together, these findings demonstrate the superiority of the proposed method.

## 1. Introduction

Analog circuits are widely used in industrial production and daily life. With the rapid development of modern electronic products, the operational reliability and maintenance assurance requirements for analog circuits have become increasingly stringent [1]. Due to factors such as defective manufacturing of electronic components, degradation of electrical performance, and external stimuli, intermittent faults frequently occur in analog circuits [2,3]. Intermittent faults are defined as non-permanent faults that last for a short duration, appear randomly, and may resolve themselves without maintenance actions [4]. From the perspective of fault behavior, intermittent faults exhibit greater randomness than permanent faults, with both their occurrence and disappearance being sudden, and their duration being very short, making diagnosis more challenging. Frequent intermittent faults may evolve into permanent circuit faults [5]. Thus, intermittent faults pose a significant threat to the safe and stable operation of analog circuits. Furthermore, in the practical use of circuits, intermittent faults are often easily confused with anomalies caused by noise, leading to many incorrect disassembles during circuit maintenance, resulting in unnecessary repair costs and presenting a major challenge for maintenance and support [6]. Therefore, accurately distinguishing noise from intermittent faults occurring at different locations in analog circuits is of great significance for improving their reliability and maintenance assurance capabilities.

Current diagnostic methods for noise and intermittent faults can be divided into two main categories: model-based methods and data-driven methods. Model-based diagnostic methods focus on the mechanisms underlying intermittent faults, with research covering circuit boards, components, component–board interconnections, connectors, microprocessor systems, avionics systems, and more [5,7]. However, as electronic products become increasingly complex and large-scale, fully understanding their internal model structures is becoming more challenging. In this context, data-driven diagnostic methods have emerged.

Data-driven methods learn features from data and use classifiers for fault diagnosis. The key to solving fault diagnosis problems with these methods lies in selecting suitable feature extraction techniques and high-performance classifiers. Currently, time–frequency analysis is the most common approach for feature extraction in intermittent fault diagnosis. These techniques can be divided into two categories: non-adaptive and adaptive methods. Non-adaptive methods, such as Wavelet Transform (WT), decompose the original signal into time–frequency components at multiple scales. Manohar et al. [8] proposed a pattern detection technique based on Discrete Wavelet Transform (DWT) and Extreme Learning Machine (ELM) for intermittent fault diagnosis in wind turbine microgrid operations. However, WT has limited resolution for high-frequency signals and is highly dependent on the selection of mother wavelet functions. Adaptive techniques, like Empirical Mode Decomposition (EMD), can decompose complex signals into intrinsic mode function components (IMFs). Liu et al. [9] used EMD with Support Vector Machine (SVM) for bearing fault diagnosis. EMD, however, suffers from mode mixing and endpoint effects, leading to the development of techniques like EEMD [1]. The selection of intrinsic mode function components significantly impacts diagnostic results. In conclusion, selecting appropriate features for intermittent fault diagnosis remains a challenging task.

The continuous development of entropy theory has introduced new approaches to feature extraction for intermittent faults. Unlike time–frequency analysis methods, entropy-based methods do not require signal decomposition but focus on measuring signal characteristics, directly quantifying their regularity. These methods are particularly suitable for nonlinear and non-stationary signals and have been widely applied in fault diagnosis. Common entropy measures in fault feature extraction include Approximate Entropy, Sample Entropy, Permutation Entropy, and Fuzzy Entropy. However, each has limitations: Approximate Entropy is sensitive to time series length and often yields underestimated values [10]; Sample Entropy addresses some issues but can give incorrect results [11]; Permutation Entropy is computationally efficient but disregards numerical data [12]; fuzzy entropy uses fuzzy membership functions, offering a more comprehensive description of signal complexity [13]. Zheng et al. [14] introduced Multiscale Fuzzy Entropy (MFE), which improves noise resistance and classification capability compared to single-scale fuzzy entropy. Key multiscale fuzzy entropy methods include composite multiscale fuzzy entropy (CMFE) [15], refined composite multiscale fuzzy entropy (RCMFE) [16], and improved multiscale fuzzy entropy (IMFE) [17]. While these methods are mainly applied in mechanical fault diagnosis, their use in analog circuit fault diagnosis remains limited. Huang et al. [18] combined Local Mean Decomposition (LMD) and Multiscale Entropy to extract intermittent fault features, utilizing deep forest for analog circuit fault diagnosis.

In summary, the MFE method stands out as a leading approach among current entropy-based methods. Although MFE has primarily been applied in the field of mechanical fault diagnosis, it excels at handling nonlinear and non-stationary signals and effectively measures the regularity of signals. These characteristics make it equally suitable for processing intermittent fault signals in analog circuits. Additionally, MFE provides fuzzy entropy results across multiple scales, enabling a more detailed representation of the differences between noise and intermittent faults. Therefore, MFE is highly suitable for feature extraction in the diagnosis of noise and intermittent faults in analog circuits.

For classifier selection, traditional machine learning classifiers, such as Support Vector Machines (SVMs), excel in small-sample and nonlinear classification tasks. Gao et al. [19] applied WT for feature extraction from analog circuit signals and used an improved SVM for fault classification. However, selecting an appropriate kernel function for SVMs remains challenging, and they are less effective for multi-class problems. Neural networks have introduced new classifiers, such as Backpropagation (BP) networks [20], but they suffer from slow convergence and local optima. Deep learning overcomes these limitations by automatically learning valuable features from data, and it was widely applied in intermittent fault diagnosis [21]. Common deep learning classifiers include Deep Belief Networks (DBNs) [1], Autoencoders [22], convolutional neural networks (CNNs) [23], Long Short-Term Memory (LSTM) networks [24], Spiking Neural Networks (SNNs) [25], and Transformers [26]. Due to their strong self-learning capabilities, deep learning methods are often directly applied for feature extraction and diagnosis, such as 1D-CNN (Yang) [27], CNN + LSTM [28], PSAL [29], and A-LSTM [30]. While deep learning methods are becoming increasingly popular, each approach has its strengths and weaknesses. DBNs are suitable for small-sample and unsupervised tasks but struggle with time series data. LSTMs handle time series well but involve complex computations and long training times. Autoencoders are better for signal reconstruction and denoising, but they have weak classification capabilities. CNNs and Transformers are both effective for time series, with CNNs focusing on local features and Transformers emphasizing global features. Therefore, classifier selection in data-driven methods depends on specific task requirements.

Intermittent faults and noise exhibit similar waveform characteristics, making them difficult to distinguish. However, their generation mechanisms are fundamentally different, revealing distinct waveforms. The similarity in waveform appearance does not obscure the differences in their underlying variation patterns. Multiscale fuzzy entropy measures signal regularity across multiple scales, emphasizing the differences between intermittent faults and noise signals. However, traditional fuzzy entropy methods do not account for normal data points in intermittent fault signals, leading to reduced entropy values and potential loss of subtle differences. Therefore, an improved fuzzy entropy calculation method is needed to mitigate the influence of normal signals. Additionally, the traditional sliding process increases the step size with scale, causing information loss in short time series signals. A finer sliding strategy is required to address this issue. Furthermore, fuzzy entropy overlooks amplitude characteristics, making it difficult to distinguish signals with similar regularities but different amplitudes. Thus, extracting amplitude features is essential to complement fuzzy entropy features for classification. Finally, a suitable classifier model is necessary to integrate these two types of features. CNNs focus on local differences, and combining multiple CNNs can provide comprehensive diagnostic results, making them an effective choice for classification.

This paper proposes a fusion method combining multiscale fuzzy entropy and amplitude features for diagnosing noise and intermittent faults in analog circuits, aiming to achieve high-accuracy classification and distinguishing noise as a single class from intermittent faults, which originate at different locations. This approach can reduce incorrect disassembly during circuit maintenance, lower repair costs, and enhance the reliability and maintenance support capabilities of the circuit. The main contributions can be summarized as follows:

1. A novel fault diagnosis method based on the fusion of multiscale fuzzy entropy features and amplitude features is proposed. Multiscale fuzzy entropy features are derived through a novel signal coarse-graining and fuzzy entropy calculations. A self-attention mechanism is employed to fuse multiple statistical characteristics of signals into comprehensive amplitude features. The proposed method integrates these features using a convolutional neural network to enhance diagnosis accuracy, achieving precise classification of noise and intermittent faults occurring at different locations.
2. For multiscale fuzzy entropy feature extraction, a multiscale fuzzy entropy calculation method with an adjustable sliding window is proposed. By adjusting the window size, normal data are excluded to offset the downward influence of normal data on fuzzy entropy calculations for intermittent faults. The process adopts a point-by-point sliding strategy, increasing the iterations to address the issue of information loss in short time sequences.
3. For amplitude feature extraction, a method based on self-attention mechanism fusion is proposed. The original signal is segmented, and features such as mean, interquartile range, Variance, and root mean square are calculated for each segment, transforming the signal into four statistical features. The self-attention mechanism fuses these statistical features into a comprehensive amplitude feature, capturing amplitude characteristics more effectively than single statistical features.

The remainder of this paper is organized as follows: Section 2 introduces the calculation process of multiscale fuzzy entropy, the challenges of applying fuzzy entropy to intermittent fault diagnosis, and the fundamental concepts of CNN and the self-attention mechanism. Section 3 proposes the method for intermittent fault diagnosis based on multiscale fuzzy entropy and amplitude features, detailing the overall structure, specific components, and parameter selection of the diagnosis process. Section 4 validates the effectiveness of the proposed diagnosis method through two typical filter circuit case studies. Section 5 concludes the paper.

## 2. Preliminaries

### 2.1. Multiscale Fuzzy Entropy

Compared to single-scale fuzzy entropy, multiscale fuzzy entropy is more suitable for handling complex time series data and exhibits stronger noise resistance. The specific calculation steps for multiscale fuzzy entropy are as follows:

(1) For a time series $x\left(n\right)=x\left(1\right),x\left(2\right),\ldots ,x\left(N\right)$ consisting of N data points, a vector sequence of dimension m can be constructed within time i, expressed as follows:
  (1) $$x_{i}^{m}=\left\{x\left(i\right),x\left(i+1\right),\ldots x\left(i+m-1\right)\right\}-x_{a}\left(i\right),\;\;\;i=1,2,\ldots ,N-m+1$$
  Here, $x_{a}(i)$ represents the mean of $x_{i}^{m}$, expressed as follows:
  (2) $$x_{a}\left(i\right)=\frac{1}{m}\sum_{k=0}^{m-1}x\left(i+k\right),\;\;\;i=1,2,\ldots ,N-m+1$$
(2) For the vector sequence $x_{i}^{m}$, the distance $d_{ij}^{m}$ between two vectors $x_{i}^{m}$ and $x_{j}^{m}$ is defined as the maximum absolute difference in their corresponding scalar components.
  (3) $$d_{ij}^{m}=d\left[x_{i}^{m},x_{j}^{m}\right]={max}_{k\in \left(0,m-1\right)}=\left\{\left|\left[u\left(i+k\right)-u_{a}\left(i\right)\right]-\left[u\left(j+k\right)-u_{a}\left(j\right)\right]\right|\right\},\;\;\;i,j=1,2,\ldots ,N-m+1,i\neq j$$
(3) The similarity between $x_{i}^{m}$ and $x_{j}^{m}$ is measured using an exponential function, expressed as follows:
  (4) $$D_{ij}^{m}=\mu \left(d_{ij}^{m},n,r\right)=e^{-ln2{\left(\frac{d_{ij}^{m}}{r}\right)}^{n}}$$
  where n and r represent the gradient and width of the boundary, respectively.
(4) Define $\phi^{m}(n,r)$, expressed as follows:
  (5) $$\phi^{m}\left(n,r\right)=\frac{1}{N-m}\sum_{i=1}^{N-m}\left[\frac{1}{N-m-1}\sum_{j=1,j\neq i}^{N-m}D_{ij}^{m}\right]$$
(5) Increase the dimension m to m+1, calculate the distance $d_{ij}^{m+1}$ between $x_{i}^{m+1}$ and $x_{j}^{m+1}$, and compute the corresponding similarity $D_{ij}^{m+1}$.
(6) Calculate $\phi^{m+1}(n,r)$, expressed as follows:
  (6) $$\phi^{m+1}\left(n,r\right)=\frac{1}{N-m}\sum_{i=1}^{N-m}\left[\frac{1}{N-m-1}\sum_{j=1,j\neq i}^{N-m}D_{ij}^{m+1}\right]$$
(7) Compute the fuzzy entropy of the time series $x\left(n\right)$, expressed as follows:
  (7) $$FuzzyEn\left(m,n,r\right)=\underset{N\to \infty }{\lim}\left[ln\phi^{m}\left(n,r\right)-ln\phi^{m+1}\left(n,r\right)\right]$$
  If N is finite, FuzzyEn(m,n,r) can also be expressed as follows:
  (8) $$FuzzyEn\left(m,n,r,N\right)=ln\phi^{m}\left(n,r\right)-ln\phi^{m+1}\left(n,r\right)$$
(8) After determining the scale factor τ, the original time series is divided into several non-overlapping windows of length τ (where τ is a positive integer). The average value within each window is calculated, forming a new data point. The sequence of these averages creates a coarse-grained time series $y_{j}^{\tau }$, expressed as follows:
  (9) $$y_{j}^{\tau }=\frac{1}{\tau }\sum_{i=\left(j-1\right)\tau +1}^{j\tau }x\left(i\right),\;1\leq j\leq \frac{N}{\tau }$$
(9) For each coarse-grained time series $y_{j}^{\tau }$, calculate the corresponding fuzzy entropy and plot it as a function of the scale factor τ.
  (10) $$MFE\left(x,\tau ,m,n,r,N\right)=FuzzyEn\left(y_{j}^{\tau },m,n,r,N\right)$$

The parameters m,n,r remain the same across all coarse-grained time series obtained using different scale factors τ.

### 2.2. Application Issues of Fuzzy Entropy in Intermittent Fault Diagnosis

In analog circuits, intermittent faults can arise due to environmental factors, mechanical stress during circuit operation, poor manufacturing processes, or long-term component degradation [31].

**Figure 1:**
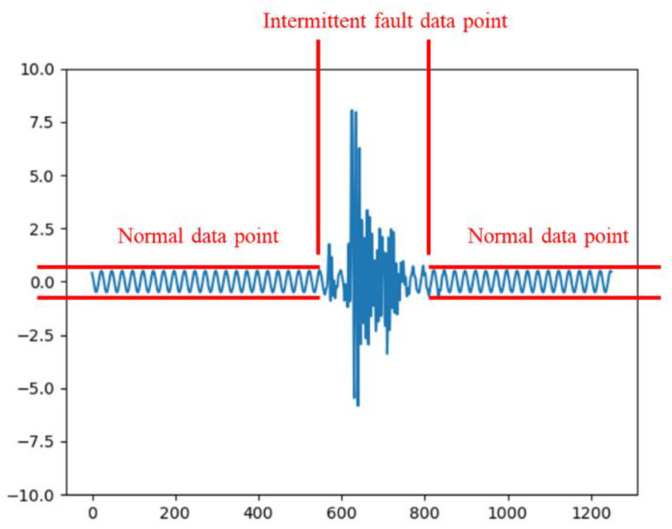
Waveform of intermittent fault.

Unlike permanent faults, intermittent faults are transient, with durations rarely exceeding 5 ms [32], and they disappear without maintenance. A typical waveform of an intermittent fault in an analog circuit is shown in Figure 1. As observed from Figure 1, intermittent fault waveforms often contain normal data points. Including these normal points in the fuzzy entropy calculation for intermittent faults reduces the computed entropy value. This effect undermines the ability of fuzzy entropy features to reflect waveform differences, hindering effective classification.

**Figure 2:**
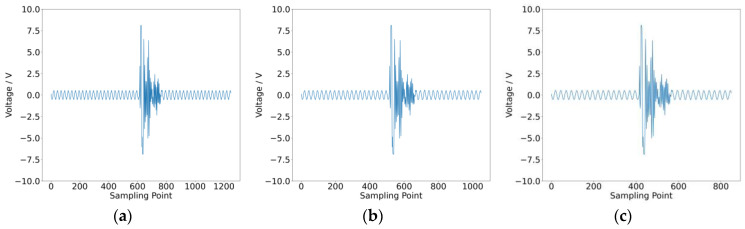

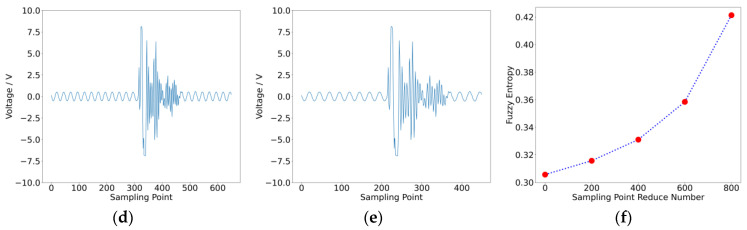
Impact of normal data points on fuzzy entropy: (**a**) 1250 points; (**b**) 1050 points; (**c**) 850 points; (**d**) 650 points; (**e**) 450 points; (**f**) fuzzy entropy result.

To validate this observation, a typical intermittent fault was tested, as shown in Figure 2. The waveform contains 1250 data points, of which approximately 200 points represent the fault. The fuzzy entropy of this waveform was calculated. Subsequently, normal data points at both ends of the waveform were truncated, with 100 points removed from each side (a total of 200 points). The entropy of the truncated waveform was recalculated. This process was repeated several times, and the results are presented in Table 1. It is evident that as more normal data points were removed, the entropy of the intermittent fault waveform increased. This is because the influence of normal points in lowering entropy values was reduced, resulting in values closer to the true entropy of the intermittent fault.

In summary, the more normal data points are removed from the intermittent fault waveform, the higher the corresponding fuzzy entropy value becomes. When all the normal data points are removed, the fuzzy entropy reaches its maximum value. However, in practical data processing, it is not always possible to remove all normal data points. Therefore, minimizing the influence of normal data points during the entropy calculation can enhance the diagnostic capability for intermittent faults.

### 2.3. Convolutional Neural Networks

**Figure 3:**
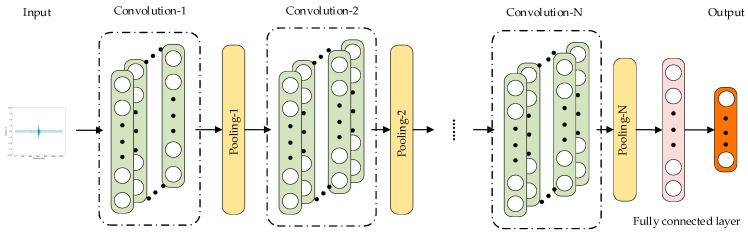
Structure of 1D-CNN.

CNNs are widely applied in fault diagnosis. The core concept of CNNs is to use convolution and pooling operations to extract and classify features from input data. As illustrated in Figure 3, a simple one-dimensional CNN typically consists of convolutional layers, pooling layers, fully connected layers, and an output layer. The convolutional layer is the core of CNNs, extracting features from input data. The pooling layer reduces the dimensionality of features, while the fully connected layer maps the features extracted by the convolutional and pooling layers to the output space. The output layer usually employs a SoftMax function for classification, producing the final results.

(11) $$P\left(y=j|x\right)=\frac{e^{z_{j}}}{\sum_{k=1}^{K}z_{k}}$$

where $P\left(y=j|x\right)$ represents the probability of the output y=j given the input x; $z_{j}$ is the j-th value of the fully connected layer output; and K is the number of output categories. In this study, we will choose CNN as the classifier.

### 2.4. Self-Attention Mechanism

The self-attention mechanism can capture global dependencies in time series data and enables efficient parallel computation, making it widely applicable in the field of fault diagnosis. The most commonly used attention mechanism is the scaled dot product attention [26]. This mechanism computes similarity by taking the dot product of query (Q) and key (K). The similarity is then divided by the scaling factor $\sqrt{d_{k}}$ to mitigate the adverse effects of unstable gradients. Finally, the attention weights are computed using the SoftMax function and multiplied with value (V).

For input features $F=\{f_{1}^{d},f_{2}^{d},f_{3}^{d},\ldots ,f_{n}^{d}\}$, where n is the number of features and d is the input dimension of each feature, three weight matrices ($W^{Q}$, $W^{K}$, $W^{V}$) are applied to calculate the query (Q), key (K), and value (V) for each feature.

(12) $$Q=W^{Q}F,\;K=W^{K}F,V=W^{V}F$$

The self-attention mechanism can be illustrated as follows:

(13) $$Attention\left(Q,K,V\right)=Softmax(\frac{QK^{T}}{\sqrt{d_{k}}})V$$

## 3. Proposed Method

### 3.1. Structure of Intermittent Fault Diagnosis Method

**Figure 4:**
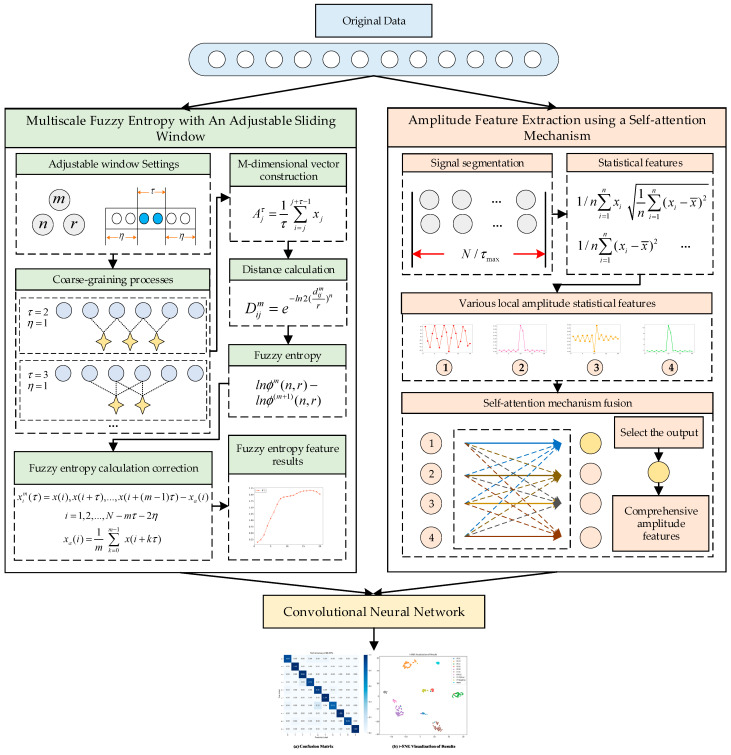
Structure of proposed method. During the coarse-graining process, the light blue small circles represent the original data, and the yellow four-pointed stars represent the results after coarse-graining. During the signal segmentation process, the gray small circles represent the original data. During the self-attention mechanism fusion process, the yellow and red small circles represent the features after fusion through the self-attention mechanism.

The structure of the proposed intermittent fault diagnosis method for analog circuits, based on the fusion of multiscale fuzzy entropy features and amplitude features, is shown in Figure 4.

The entire model consists of three parts: multiscale fuzzy entropy feature extraction with an adjustable sliding window, amplitude feature extraction using a self-attention mechanism, and a convolutional neural network. In the fuzzy entropy feature extraction module, normal data points are excluded by the adjustable sliding window to reduce their impact on the fuzzy entropy calculation. A point-by-point sliding method is used to prevent information loss. In the amplitude feature extraction module, the signal is converted into four amplitude statistical features. These features are fused using a self-attention mechanism, and the output corresponding to the mean value is selected as the final amplitude feature. By integrating multiple statistical perspectives, the comprehensive feature reflects the amplitude characteristics of the signal more effectively than single statistical features. Finally, the entropy features and amplitude features are fed into two separate convolutional networks. Each convolutional network performs convolutional computations to extract convolutional features. The output convolutional features from both networks are then concatenated and passed through a fully connected layer to produce the classification results.

### 3.2. Multiscale Fuzzy Entropy with Adjustable Sliding Window

**Figure 5:**
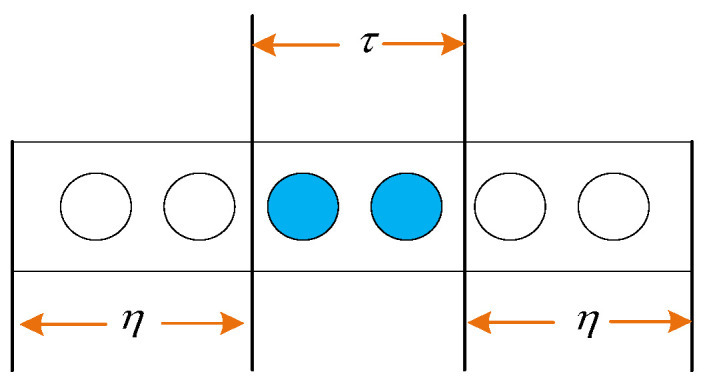
Adjustable sliding window.

To address the influence of normal data points on the fuzzy entropy calculation in traditional multiscale fuzzy entropy and the issue of information loss when dealing with short time series data, this study proposes a multiscale fuzzy entropy calculation method with an adjustable sliding window. The proposed method involves the following three steps:

(1) Determine the adjustable sliding window, as shown in Figure 5. The adjustable sliding window consists of a regulation module and a scale module. The regulation module, with a length of η, determines the length of data to be excluded from entropy calculation, thereby reducing the influence of normal waveform data on the entropy calculation of intermittent faults. The scale module, with a length of τ, performs the coarse-graining process.

Assuming the data sampling frequency is f and the sampling time is t, the sliding window is given by the following:

(14) η=λft

where λ is the adjustable factor.

**Figure 6:**
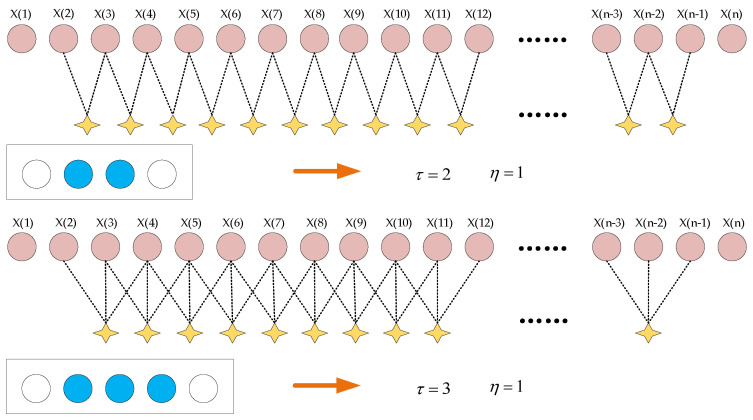
Coarse-graining process of proposed method.

(2) Perform coarse-graining on the original time series. As shown in Figure 6, for each scale factor τ, the predefined adjustable sliding window begins sliding from the first data point, with a step size of 1. During sliding, data points excluded by the regulation module of length η are ignored, and the average of τ data points is calculated to form a new data point. This process generates a coarse-grained time series.
  (15) $$A_{j}^{\tau }=\frac{1}{\tau }\sum_{i=j}^{j+\tau -1}x_{i},\eta +1\leq j\leq N-2\eta -\tau +1$$
  where N and τ represent the length of the original time series and the scaling factor, respectively; and η is the length of the regulation module. Using Equation (15), the original time series $x_{i}$ is divided into a coarse-grained vector sequence $A^{\tau }$, with a length of N−2η−τ+1.

(3) For the newly constructed vector sequence $A^{\tau }$, fuzzy entropy can be computed. At this stage, the original fuzzy entropy formula is modified based on the correction strategy referenced in [17]. The correction introduces the scaling factor τ to account for time delays. For a time series $x\left(n\right)=x\left(1\right),x\left(2\right),\ldots ,x\left(N\right)$ with N data points, a vector sequence of dimension mmm can be constructed at time i, expressed as follows:
  (16) $$x_{i}^{m}\left(\tau \right)=\left\{x\left(i\right),x\left(i+\tau \right),\ldots x\left(i+\left(m-1\right)\tau \right)\right\}-x_{a}\left(i\right),\;\;\;i=1,2,\ldots ,N-m\tau -2\eta$$

Here, $x_{a}(i)$ represents the mean of $x_{i}^{m}\left(\tau \right)$, expressed as follows:

(17) $$x_{a}\left(i\right)=\frac{1}{m}\sum_{k=0}^{m-1}x\left(i+k\tau \right)$$

The subsequent formulas for $D_{ij}^{m}$, $\phi^{m}\left(n,r,\eta ,\tau \right)$, $\phi^{m+1}\left(n,r,\eta ,\tau \right)$, and $FuzzyEn\left(m,n,r,\eta ,\tau \right)$ remain unchanged.

### 3.3. Parameter Determination

The proposed method involves six parameters: m,r,n,τ,λ,η. The parameter m represents the size of the observation window for the original sequence, r is the threshold for window determination, n denotes the gradient of the window, τ corresponds to different scales in the coarse-graining process, and λ is the adjustable factor used to control the length of the discarded data η. Based on previous studies [14], the recommended values for the first four parameters are m=2, r=0.15 std (std represents the standard deviation of the original time series), n=2, and τ ranging from 1 to 20.

λ and η are determined as follows:

In the actual process of intermittent fault signal acquisition, the collected signals often contain normal data points. The presence of the adjustable factor λ helps to eliminate these normal data points, thereby enhancing the classification capability of the calculated fuzzy entropy results. Assuming that the collected signal contains a total of k data points, the position of the first data point is set to 1, the position of the second data point is set to 2, and so on; the position of the last data point is set to k. If the position of the data point that exceeds the normal data threshold for the first time in the signal is $k_{s}$, the position of the data point that exceeds the normal data threshold for the last time is $k_{e}$. At this point, we can calculate the following:

(18) $$\lambda_{s}=\frac{k_{s}-1}{k}$$

(19) $$\lambda_{e}=\frac{k-k_{e}}{k}$$

The value of λ is determined to be the following:

(20) $$\lambda =\min\;(\lambda_{s},\;\lambda_{e})$$

The value of η is determined by Formula (14). If multiple pieces of data are included in the calculation, calculate $\lambda_{s}$ and $\lambda_{e}$ for each piece of data separately, and choose λ as the smallest value of all $\lambda_{s}$ and $\lambda_{e}$. The aforementioned parameter determination method can be used to compute a universal adjustable factor λ.

### 3.4. Amplitude Feature Extraction Using Self-Attention Mechanism

Multiscale fuzzy entropy features focus on measuring the regularity of signals and can distinguish noise and intermittent fault signals to a certain extent. However, after signal transformation using the fuzzy function, amplitude information is lost. This limitation makes multiscale fuzzy entropy features ineffective in distinguishing signals with similar regularities but significant amplitude differences. Therefore, extracting amplitude features that reflect signal variations and using them to enhance fuzzy entropy features can further improve diagnostic performance.

**Figure 7:**
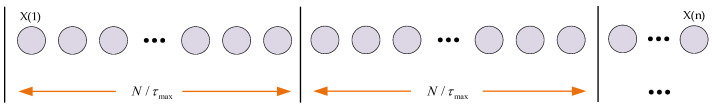
Segmentation for amplitude feature extraction.

To better capture the amplitude variations in the signal and align the length of amplitude features with fuzzy entropy features for easier classifier design, this study segments the time series signal using the maximum scale factor $\tau_{max}$ from the fuzzy entropy calculation process. Subsequently, the mean, interquartile range (IQR), Variance, and root mean square (RMS) of each segment are computed, converting the signal into four amplitude statistical features. The segmentation process is shown in Figure 7, and the formulas for the statistical features are summarized in Table 2.

**Figure 8:**
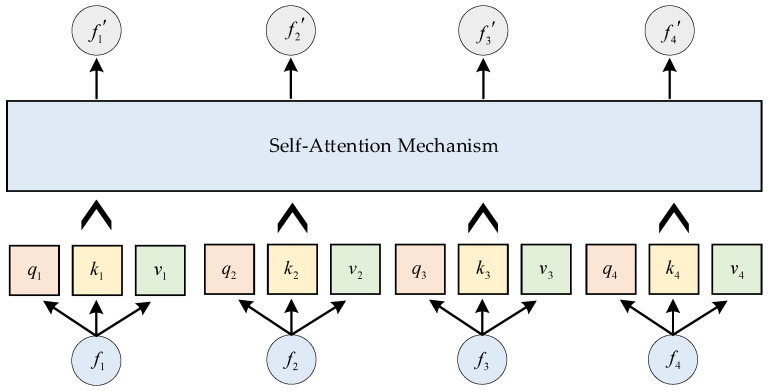
Self-attention mechanism fusion process.

After obtaining the four statistical features, a self-attention mechanism is introduced for feature fusion. In the self-attention mechanism, given 4 input features F, the algorithm produces 4 corresponding fused results $F^{\prime }$. As shown in Figure 8, taking $f_{1}^{\prime }$ as an example, its computation process can be expressed as follows:

(21) $$f_{1}^{\prime }=\sum_{i=1}^{4}Softmax(\frac{q_{1}k_{i}^{T}}{\sqrt{d_{k}}})v_{i}$$

In this study, the differences among the four output features obtained through the self-attention mechanism primarily stem from the differences in query (Q). Therefore, there is a one-to-one correspondence between the input features F and the output features $F^{\prime }$. In the self-attention computation process, the feature from which the query originates indicates that this computation is focused on the feature learning information related to itself from both itself and other features. Among the four statistical features calculated in this study, the mean is the most sensitive to abnormal variations in the signal.

Therefore, instead of inputting all four output features into the subsequent classification model, we select the output feature corresponding to the mean as the comprehensive amplitude feature for further classification. The weight matrices associated with the comprehensive feature are learned and optimized through the classification model.

### 3.5. The Convolutional Neural Network Structure in the Proposed Method

**Figure 9:**
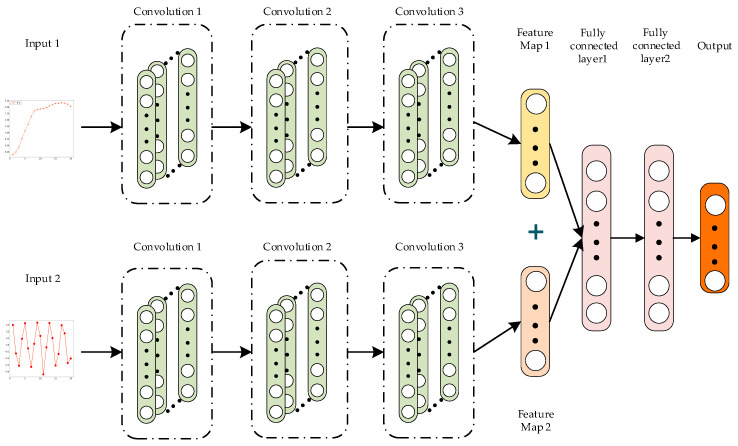
Convolutional neural network.

The obtained multiscale fuzzy entropy features and comprehensive amplitude features are used as inputs to train the convolutional neural network. As shown in Figure 9, we designed a convolutional neural network comprising two convolutional modules.

The inputs to these modules are the multiscale fuzzy entropy features and the comprehensive amplitude features, respectively. Pooling layers typically help reduce overfitting and improve the generalization ability of convolutional neural networks. However, in this study, the input data size is small, and using a pooling layer may lead to excessive dimensionality reduction, resulting in significant information loss that impacts network performance. Therefore, the network structure designed in this study does not include a pooling layer. Each convolutional module comprises three convolutional layers. After passing through these three layers, the input data generates two convolutional features, which are directly concatenated. Finally, a fully connected layer is used to output the classification results. This network structure is simple, trains quickly, and is highly suitable for the fusion and classification of multiscale fuzzy entropy features and comprehensive amplitude features. The specific network structure parameters are shown in Table 3.

## 4. Experiment Results and Comparison

### 4.1. Experimental Circuit Setup

We selected two types of active filter circuits as case studies: a Sallen–Key bandpass filter circuit and a quad operational amplifier dual-fourth-order high-pass filter circuit. These circuits are highly representative in studying the problem of intermittent fault diagnosis in analog circuits [22,32].

(1) Case 1—Sallen–Key bandpass filter circuit

**Figure 10:**
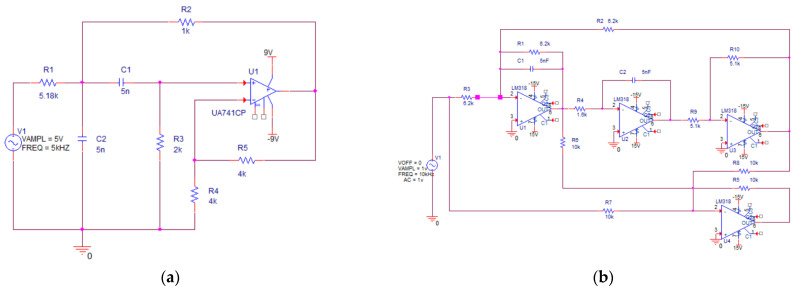
Experimental circuit. (**a**) Sallen–Key bandpass filter circuit; (**b**) quad operational amplifier dual-fourth-order high-pass filter circuit.

**Figure 11:**
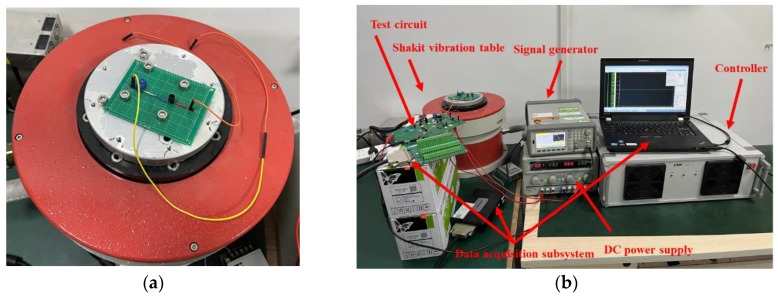
(**a**) Intermittent fault excitation device for Case 1; (**b**) shock test system for Case 1.

Case 1 is a shock test. Figure 10a shows the circuit diagram and associated component parameters of the Sallen–Key bandpass filter circuit. Physical impact experiments were conducted to collect intermittent fault data and normal waveform data for the Sallen–Key bandpass filter circuit. Figure 11a illustrates the fault-triggering setup for the shock test. The fault-triggering setup consists of a Shakit vibration table and an adjustable terminal block. The Shakit vibration table provides an external vibration environment to stimulate intermittent fault occurrences. The terminal block is connected with wires in series to different components of the Sallen–Key bandpass filter circuit, effectively introducing a series resistor at various positions in the circuit to simulate solder joint intermittent faults. The resistance value of this series resistor changes with the tightness of the terminal block. A looser terminal block corresponds to a higher series resistance, which in turn simulates a more severe intermittent fault. The shock test system, as shown in Figure 11b, includes a bandpass filter circuit, a DC power supply, a signal generator, a controller, an intermittent fault excitation device, and a data acquisition subsystem containing a USB3122 data acquisition board and a computer.

The terminal block was connected to various components of the Sallen–Key bandpass filter circuit, including R1, C1, C2, R2, R3, R4, R5, the positive terminal of the operational amplifier, and the negative terminal of the operational amplifier. This setup simulates nine types of intermittent faults at different locations. For each location, the terminal block’s tightness was adjusted to two states: a loosening of 0.25 turns and 0.5 turns from the normal state. The rotation angles were strictly controlled by monitoring the rotation degrees. Intermittent faults were triggered using the Shakit vibration table and its controller, with precise control over the start and end times of vibration through software. The fault-triggering duration was controlled within 5 ms. To better capture intermittent fault data, the sampling frequency was set to 250 kHz. Each position and each tightness state underwent 150 experiments, resulting in a total of 2700 experimental datasets. Noise data were generated by adding Gaussian noise to normal waveform data. The duration and severity of the added noise were entirely random.

(2) Case 2—Quad operational amplifier dual-fourth-order high-pass filter circuit

Case 2 is a simulation case. Figure 10b shows the circuit diagram and associated component parameters of the quad operational amplifier dual-fourth-order high-pass filter circuit. The circuit includes a switching device to trigger intermittent faults. The sensitive components in this circuit are C2, R3, and R4 [18]. Intermittent faults were injected into these sensitive components. The circuit simulation was conducted using Pspice 17.4 software. The input signal for the circuit was a 1 V, 10 kHz AC voltage. The resistance tolerance was set to 10%, and the sampling frequency was set to 250 kHz. Each component underwent 150 experiments, yielding a total of 450 experimental datasets.

In Case 1, nine intermittent faults at different locations and random noise were setup. All noise was grouped into one class, resulting in ten classification labels in total. In Case 2, three intermittent faults at different locations and random noise were setup. Similarly, all noise was grouped into one class, resulting in a total of four classification labels. Detailed information about the classification labels is provided in Table 4.

### 4.2. Waveform Data and Diagnostic Results

**Figure 12:**
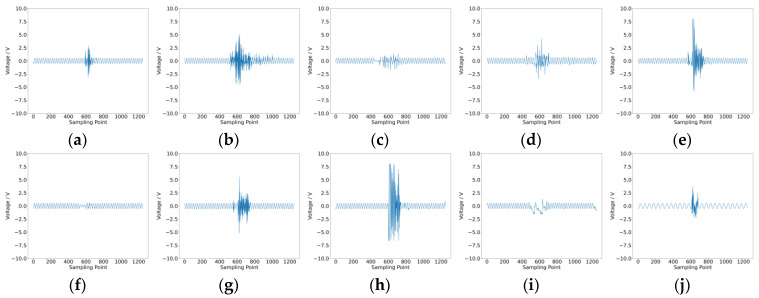
Data waveform for Case 1. (**a**) Intermittent fault R1; (**b**) intermittent fault C2; (**c**) intermittent fault C1; (**d**) intermittent fault R2; (**e**) intermittent fault R3; (**f**) intermittent fault R4; (**g**) intermittent fault R5; (**h**) intermittent fault positive; (**i**) intermittent fault negative; (**j**) noise.

**Figure 13:**
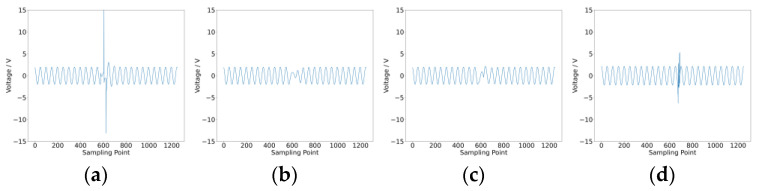
Data waveform for Case 2. (**a**) Intermittent fault C2; (**b**) intermittent fault R3; (**c**) intermittent fault R4; (**d**) noise.

After collecting intermittent fault and noise data from the two cases, we proceeded to extract fuzzy entropy features and amplitude features. The waveform data for intermittent faults and noise in Case 1 are shown in Figure 12, and those for Case 2 are shown in Figure 13. Both types of features were input into the convolutional neural network for training and testing. Each case was trained five times, with no fewer than 600 epochs per training session.

**Figure 14:**
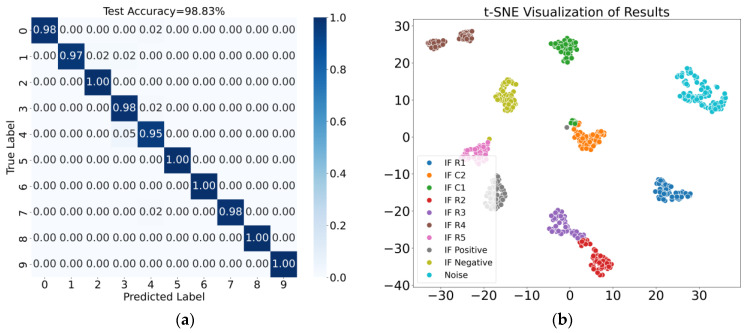
Results for Case 1. (**a**) Confusion matrix; (**b**) t-SNE visualization of results.

**Figure 15:**
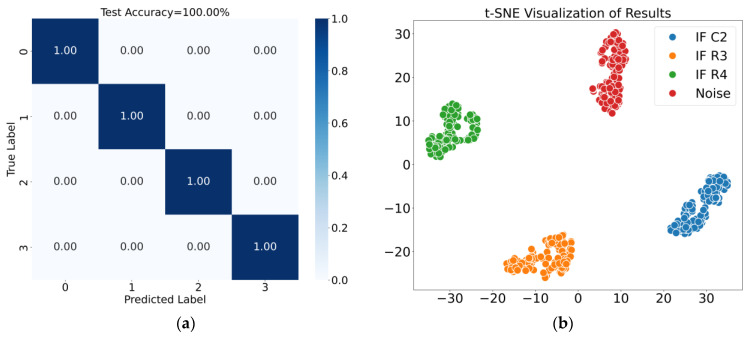
Results for Case 2. (**a**) Confusion matrix; (**b**) t-SNE visualization of results.

The average classification accuracy was used as the evaluation metric. The confusion matrix and t-SNE visualization results for Case 1 are shown in Figure 14, where the fusion features achieved an average classification accuracy of 98.83%. Similarly, the results for Case 2 are shown in Figure 15, with the fusion features achieving an average classification accuracy of 100.00%. These diagnostic results demonstrate that the proposed method effectively distinguishes noise and intermittent faults occurring at different locations.

By performing a horizontal comparison between Case 1 and Case 2, we found that Case 2 achieved a higher average classification accuracy, reaching 100%. This is mainly due to the different ways in which intermittent faults were triggered in the two cases. In Case 1, intermittent faults were triggered by changing the contact resistance and applying external stimuli. The design of the intermittent fault excitation device was more representative of real-world conditions, so the experimental data obtained were more objective and complex, making the classification task more challenging. In contrast, in Case 2, intermittent faults were triggered by controlling the circuit connection and disconnection with a high-speed switch. The design of the intermittent fault excitation device was simpler and more convenient, leading to relatively simpler experimental data, which made the classification task easier compared to Case 1.

### 4.3. Effectiveness of Multiscale Fuzzy Entropy Features with Adjustable Sliding Window

To verify the effectiveness of multiscale fuzzy entropy features with an adjustable sliding window, we compared the proposed method with four mainstream multiscale fuzzy entropy calculation methods currently used in the diagnosis field. These methods were used to extract fuzzy entropy features: MFE [14], CMFE [15], RMFE [16], IMFE [17], and the proposed method. A CNN classifier was employed. Each combination was trained five times, and the average classification accuracy was used as the evaluation metric. The classification results for both cases are shown in Table 5.

As observed from Table 5, in both cases, the fuzzy entropy features extracted using the proposed method achieved the highest average classification accuracy, reaching 94.15% and 99.17%, respectively. This indicates that the proposed fuzzy entropy feature extraction method is more suitable for distinguishing noise and intermittent faults at different locations. The new method eliminates normal data points, reducing their downward influence on the fuzzy entropy calculation. Additionally, the point-by-point smoothing strategy avoids information loss, further enhancing its effectiveness.

### 4.4. Effectiveness of Amplitude Feature Extraction Using Self-Attention Mechanism

To validate the effectiveness of amplitude features derived using the self-attention mechanism, we designed two ablation experiments.

(1) Comparison with single amplitude features

We selected individual features such as the mean, IQR, Variance, and RMS to compare against the comprehensive amplitude features. The goal was to verify whether the comprehensive amplitude features exhibit stronger classification capability than single amplitude features. The input consisted of each type of amplitude feature, and a CNN classifier was used. Each method combination was trained five times, and the average classification accuracy was used as the evaluation metric. The classification results for the two cases are shown in Table 6.

From Table 6, it can be observed that the comprehensive amplitude features achieved the highest average classification accuracy in both cases, reaching 87.72% and 100.00%, respectively. This demonstrates that comprehensive amplitude features outperform single amplitude features in classification. The reason is that comprehensive amplitude features consider multiple statistical perspectives, providing a more holistic reflection of the data’s amplitude characteristics.

(2) Comparison of various comprehensive amplitude features

We used the comprehensive features corresponding to the mean, IQR, Variance, and RMS as inputs individually, as well as the mean-pooling result of all four comprehensive features. The goal was to verify whether the comprehensive feature corresponding to the mean exhibited the strongest classification capability among all comprehensive features. The input consisted of each type of comprehensive amplitude feature, and a CNN classifier was selected. Each method combination was trained five times, and the average classification accuracy was used as the evaluation metric. The classification results for the two cases are shown in Table 7.

From Table 7, it is evident that the comprehensive amplitude feature corresponding to the mean achieved the highest average classification accuracy in both cases, reaching 87.72% and 100.00%, respectively. Compared to the IQR, Variance, RMS, and mean-pooling features, the mean feature better captures variations in the signal amplitude. This is further supported by Table 5, where the mean feature also demonstrated the highest classification accuracy among single amplitude features.

### 4.5. Effectiveness of Feature Fusion

To evaluate the effectiveness of feature fusion, we compared the adjustable sliding window multiscale fuzzy entropy features, the amplitude features derived using the self-attention mechanism, and their fusion. The goal was to verify whether the fusion features exhibited stronger classification capabilities than single features. A CNN classifier was employed, and each combination was trained five times. The average classification accuracy was used as the evaluation metric. The classification results for both cases are shown in Table 8.

From Table 8, it can be observed that in both cases, the fusion results of the two types of features proposed in this study achieve the highest average classification accuracy, reaching 98.83% and 100.00%, respectively. This validates two conclusions. First, the fusion of fuzzy entropy features and amplitude features exhibits stronger classification capability than individual features, demonstrating that the fusion features is effective in improving classification accuracy. Second, the combination of fuzzy entropy features and the mean-based amplitude features shows the best classification performance, confirming the effectiveness of the proposed method.

Additionally, we compared the impact of the pooling layer in the CNN network structure on the final classification results. Two CNN networks were constructed: one with the network structure proposed in this study and another that included three pooling layers. Each pooling layer has a kernel size of 2 and a stride of 2. Both networks used fused features as input and were trained five times, with average classification accuracy as the evaluation metric. As shown in Table 9, the network with pooling layers achieved average classification accuracies of 95.18% and 99.17% for Case 1 and Case 2, respectively, while the proposed network achieved 98.83% and 100.00% for Case 1 and Case 2. The results indicate that, for the small input data size in this study, the pooling layers negatively impacted the classification results, and the network without pooling layers achieved higher classification accuracy.

### 4.6. Comparison with Other State-of-the-Art Methods

Finally, the proposed method was compared with other state-of-the-art methods in the field of intermittent fault diagnosis to verify its superiority. Since the proposed method is data-driven, comparisons were primarily made with other recent data-driven methods, including MSECTN [26], 1D-CNN (Yang) [27], CNN + LSTM [28], PSAL [29], and WPD + CNN [33]. Each model was trained with a learning rate of 0.001 using the Adam optimizer for no fewer than 800 epochs. Each method was trained five times, and the average classification accuracy was used as the evaluation metric. The classification accuracy results for each model are shown in Table 10. From Table 10, it can be observed that the proposed diagnostic method, based on the fusion of multiscale fuzzy entropy features and amplitude features, achieves the highest classification accuracy compared to other state-of-the-art diagnostic methods in both Case 1 and Case 2, with accuracies of 98.83% and 100.00%, respectively.

**Figure 16:**
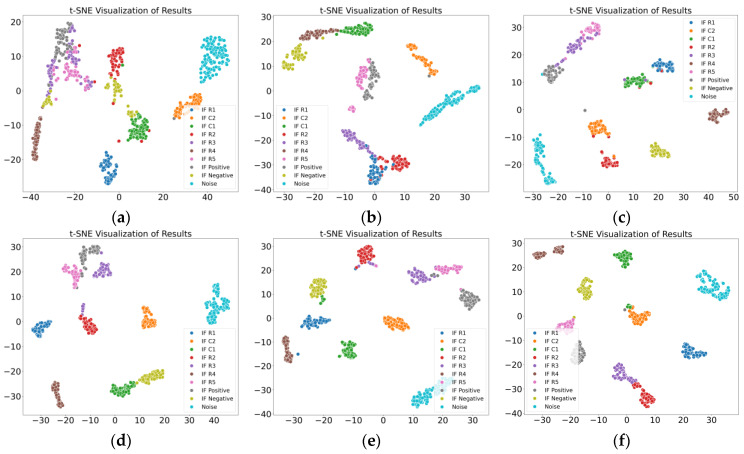
Results of various methods in Case 1. (**a**) MSECTEN; (**b**) WPD + CNN; (**c**) PSAL; (**d**) 1D-CNN (Yang); (**e**) CNN + LSTM; (**f**) ours.

**Figure 17:**
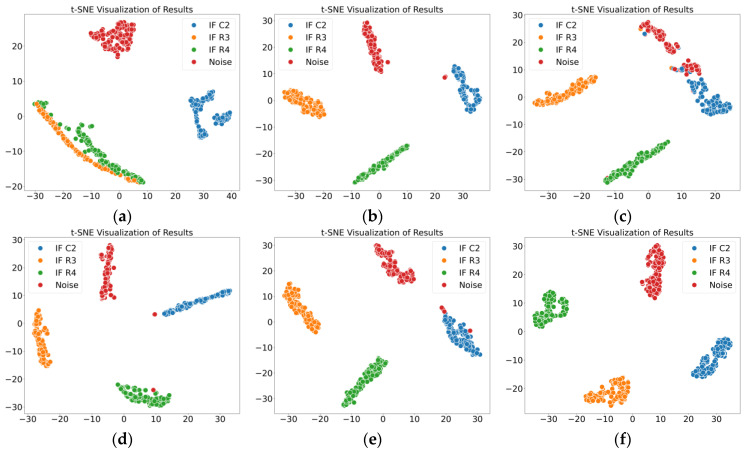
Results of various methods in Case 2. (**a**) MSECTEN; (**b**) WPD + CNN; (**c**) PSAL; (**d**) 1D-CNN (Yang); (**e**) CNN + LSTM; (**f**) ours.

The multiscale fuzzy entropy features extracted in this study represent the regularity characteristics of the signal. By incorporating an adjustable sliding window, normal data points are eliminated, reducing their negative impact on fuzzy entropy calculation. The point-by-point sliding strategy employed in the coarse-graining process mitigates signal loss, resulting in fuzzy entropy features with stronger classification capability. In addition, the study employs a self-attention mechanism to fuse the extracted amplitude features of the signal. Compared to single amplitude features, the comprehensive results derived from multiple statistical perspectives better reflect the amplitude characteristics of the signal. Finally, a convolutional neural network is used to effectively integrate multiscale fuzzy entropy features and amplitude features, further enhancing the proposed method’s ability to classify noise and intermittent faults at different locations. Thus, the proposed method demonstrates superior classification accuracy compared to other state-of-the-art methods, and the comparative results validate its effectiveness. The t-SNE visualizations of the classification results for the two cases are shown in Figure 16 and Figure 17.

Additionally, we compared the “Time to Detection” and “Diagnostic Cost” for intermittent fault diagnosis across different methods. The model training time was chosen as the indicator for diagnostic cost, while the model testing time was selected as the indicator for time to detection. All models were tested under the same hardware and software conditions, with 800 training epochs, and identical training and testing data. The processor used was the 12th Gen Intel(R) Core (TM) i7-12700KF, 3.60 GHz, and the graphics card was the NVIDIA GeForce RTX 4060Ti (16GB). The software used was Python 3.9.19. The results are shown in Table 11 and Table 12. The results indicate that 1D-CNN (Yang), CNN + LSTM, and the proposed method all demonstrate lower diagnostic costs and faster detection times. Although the proposed method does not have the lowest diagnostic cost and the fastest detection time, the differences with the two methods mentioned above are minimal, and the proposed method achieves higher classification accuracy. Taken together, these findings demonstrate the superiority of the proposed method.

## 5. Conclusions

Intermittent faults are challenging to diagnose due to their short duration, completely random occurrence, and tendency to be confused with noise. To address the high-precision classification of noise and intermittent faults occurring at different locations in analog circuits, this study proposes a fault diagnosis method based on the fusion of multiscale fuzzy entropy features and amplitude features. A series of ablation experiments validated the effectiveness of the proposed method. Furthermore, we compared it with five other state-of-the-art methods, evaluating metrics including average classification accuracy, diagnostic cost, and detection time. In terms of average classification accuracy, the proposed method achieves the highest accuracy, reaching 98.83% and 100.00% for two typical analog circuit cases. Regarding diagnostic cost, although the proposed method is not the lowest, the difference from the best-performing method is minimal in both cases, with discrepancies of 27 s and 29 s, respectively. In terms of detection time, the proposed method is not the fastest, but the difference from the best-performing method is again small, with a gap of 6 s and 7 s, respectively. In conclusion, while the proposed method does not achieve the lowest diagnostic cost or the fastest detection time, its differences from the best-performing methods are minimal, and it achieves higher classification accuracy. These findings demonstrate the superiority of the proposed method.

Therefore, this method effectively addresses the challenge of high-precision classification of noise and intermittent faults occurring at different locations in analog circuits, helping to reduce incorrect disassembly during maintenance, lower maintenance costs, and improve the reliability and maintenance support of analog circuits. The main conclusions from the experimental validation are as follows:

1. The experimental results indicate that the proposed method effectively extracts fuzzy entropy features and amplitude features. The multiscale fuzzy entropy features measure the regularity of the signal, and the amplitude features can compensate for the shortcoming of ignoring the change in signal amplitude during the calculation of the entropy features. The fusion of these two features effectively improves the diagnostic accuracy.
2. The multiscale fuzzy entropy calculation method, based on an adjustable sliding window, eliminates normal data points, reducing their negative impact on fuzzy entropy calculations. The point-by-point sliding strategy also mitigates the issue of signal loss, thereby enhancing the classification capability of fuzzy entropy.
3. The amplitude feature extraction method, based on a self-attention mechanism, integrates multiple amplitude features of the signal. Compared to single statistical features, the integration of comprehensive features from multiple statistical perspectives better reflects the amplitude characteristics of the signal, resulting in superior classification performance.

However, due to experimental limitations and the design considerations outlined, we are currently unable to compare the experimental results of each case with the simulation results. In future studies, we will further explore entropy features and attempt to propose new entropy calculation functions tailored to the characteristics of intermittent faults. Also, we plan to test different feature fusion strategies to evaluate their impact on classification performance and aim to propose improved feature fusion models.

## Figures and Tables

**Table 1:** Results of fuzzy entropy calculation.

No.	Points Lost	Fuzzy Entropy
1	0	0.3056
2	200	0.3157
3	400	0.3310
4	600	0.3584
5	800	0.4213

**Table 2:** Calculation formulas for various amplitude statistical features.

No.	Class	Formula
1	Mean	$\frac{1}{n}\sum_{i=1}^{n}x_{i}$
2	IQR	$\frac{1}{2}\left(Q_{2}+Q_{4}\right)-\frac{1}{2}(Q_{1}+Q_{3})$
3	Variance	$\frac{1}{n}\sum_{i=1}^{n}{{(x}_{i}-\bar{x})}^{2}$
4	RMS	$\sqrt{\frac{1}{n}\sum_{i=1}^{n}{{(x}_{i}-\bar{x})}^{2}}$

**Table 3:** The specific network structure parameters.

Network Layer	Convolution Kernel/Step Size (Or Other Parameters)	Number of Convolution Kernels	Output Size
Convolutional layer1	3 × 1/1 × 1	16	16 × 20
BatchNorm	-	16	16 × 20
Convolutional layer2	3 × 1/1 × 1	32	32 × 20
BatchNorm	-	32	32 × 20
Convolutional layer3	3 × 1/1 × 1	64	64 × 20
BatchNorm	-	64	64 × 20
Fully connected layer1	Number: 2560	-	2560 × 1
Dropout layer	Forgetting rate: 0.5	-	
Fully connected layer2	Number: 256	-	256 × 1
SoftMax layer	Number: 10/4	-	10/4

**Table 4:** Detailed information about classification labels.

Case Number	States	Labels	Case Number	States	Labels
Case 1	IF R1	0	Case 2	IF C2	0
	IF C2	1		IF R3	1
	IF C1	2		IF R4	2
	IF R2	3		Noise	3
	IF R3	4		/	/
	IF R4	5		/	/
	IF R5	6		/	/
	IF Positive	7		/	/
	IF Negative	8		/	/
	Noise	9		/	/

**Table 5:** Classification accuracy results for different entropy calculation methods.

Case		Case 1	Case 2
Classifier		CNN	CNN
Average classification accuracy	MFE [14]	88.89%	96.67%
	RCMFE [15]	88.30%	99.17%
	CMFE [16]	91.81%	99.17%
	IMFE [17]	94.15%	97.50%
	**Ours**	**94.15%**	**99.17%**

**Table 6:** Classification accuracy results for different single amplitude features.

Case		Case 1	Case 2
Classifier		CNN	CNN
Average classification accuracy	Only Mean	84.80%	98.33%
	Only IQR	71.93%	99.17%
	Only Variance	80.70%	98.33%
	Only RMS	82.46%	99.17%
	**Comprehensive amplitude features**	**87.72%**	**100.00%**

**Table 7:** Classification accuracy results for different comprehensive amplitude features.

Case		Case 1	Case 2
Classifier		CNN	CNN
Average classification accuracy	IQR-based features	80.70%	99.17%
	Variance-based features	83.04%	98.33%
	RMS-based features	85.96%	100.00%
	Mean-pooling features	84.80%	99.17%
	**Comprehensive amplitude features**	**87.72%**	**100.00%**

**Table 8:** Classification accuracy results for different features.

Case		Case 1	Case 2
Classifier		CNN	CNN
Average classification accuracy	Fuzzy entropy features	94.15%	99.17%
	Comprehensive amplitude features	87.72%	100.00%
	Fuzzy entropy + IQR-based	95.91%	99.17%
	Fuzzy entropy + Variance-based	96.49%	99.17%
	Fuzzy entropy + RMS-based	97.08%	100.00%
	Fuzzy entropy + mean-pooling	97.08%	100.00%
	**Ours**	**98.83%**	**100.00%**

**Table 9:** Classification accuracy results for different network structures.

Classifier		CNN	CNN with Pooling Layers
Average classification accuracy	Case 1	**98.83%**	95.18%
	Case 2	**100.00%**	99.17%

**Table 10:** Classification accuracy results for various methods.

No.	Methods	Average Classification Accuracy for Case 1	Average Classification Accuracy for Case 2
1	MSECTN [26]	90.64%	99.17%
2	WPD + CNN [33]	94.15%	99.17%
3	PSAL [29]	95.91%	98.33%
4	1D-CNN (Yang) [27]	97.08%	99.17%
5	CNN + LSTM [28]	97.66%	99.17%
6	**Our Method**	**98.83%**	**100.00%**

**Table 11:** Diagnostic cost and detection time of different methods in Case 1.

No.	Methods	Training Time for Case 1 (Diagnostic Cost)	Testing Time for Case 1 (Time to Detection)
1	MSECTN [26]	41 min	937 ms
2	WPD + CNN [33]	2 min 33 s	104 ms
3	PSAL [29]	47 min	824 ms
4	1D-CNN (Yang) [27]	1 min 31 s	75 ms
5	CNN + LSTM [28]	1 min 56 s	80 ms
6	**Our Method**	**1 min 58 s**	**81 ms**

**Table 12:** Diagnostic cost and detection time of different methods in Case 2.

No.	Methods	Training Time for Case 2 (Diagnostic Cost)	Testing Time for Case 2 (Time to Detection)
1	MSECTN [26]	28 min 59 s	534 ms
2	WPD + CNN [33]	1 min 56 s	72 ms
3	PSAL [29]	32 min 20 s	433 ms
4	1D-CNN (Yang) [27]	1 min 11 s	65 ms
5	CNN + LSTM [28]	1 min 19 s	48 ms
6	**Our Method**	**1 min 40 s**	**55 ms**

## Data Availability

Some or all data, models, or code generated or used during the study are available from the first author and the corresponding author by request.
